# Safety evaluation of fixed‐dose nivolumab in patients with gastric cancer

**DOI:** 10.1002/hsr2.673

**Published:** 2022-06-03

**Authors:** Yusuke Iikura, Takeshi Aoyama, Makoto Hiraide, Takeru Wakatsuki, Izuma Nakayama, Mariko Ogura, Akira Ooki, Daisuke Takahari, Keisho Chin, Kensei Yamaguchi, Toshihiro Hama

**Affiliations:** ^1^ Department of Pharmacy Cancer Institute Hospital, Japanese Foundation for Cancer Research Koto City Tokyo Japan; ^2^ Division of Applied Pharmaceutical Education and Research Hoshi University Shinagawa‐Ku Tokyo Japan; ^3^ Department of Gastroenterological Chemotherapy Cancer Institute Hospital, Japanese Foundation for Cancer Research Koto City Tokyo Japan

**Keywords:** fixed dose, gastric cancer, immune‐related adverse event, nivolumab, programmed death receptor‐1

## Abstract

**Background and Aims:**

This study aimed to examine the safety of fixed‐dose nivolumab.

**Methods:**

We retrospectively reviewed the medical records of 113 Japanese patients with gastric cancer who were previously treated with cytotoxic chemotherapy and initiated nivolumab. The endpoints were the incidence of Grade 2 or higher immune‐related adverse events (irAEs) in the conventional dose (3 mg/kg) and fixed‐dose groups (240 mg).

**Results:**

The incidence rates of irAEs in the conventional‐dose and fixed‐dose groups were 29.9% and 19.4%, respectively, and the rates of Grade 2 or higher irAEs were 23.3% and 19.4%, respectively, with no significant difference between the two groups, suggesting that nivolumab at 240 mg is as safe as the 3 mg/kg dose.

**Conclusion:**

This is the first report on the safety of nivolumab at 240 mg in Japanese patients.

## 1 INTRODUCTION

The effects of nivolumab, a monoclonal antibody targeting programmed death receptor‐1, are independent of body weight; hence, a fixed‐dose comparative study is appropriate to investigate its efficacy and safety.^1^ The maximum‐tolerated dose (MTD) of nivolumab is 10 mg/kg, and the maximum blood concentration that can be attained is 3 mg/kg.^2^, ^3^, ^4^, ^5^

Nivolumab has been approved as a single dose of 2 mg/kg for malignant melanoma and a single dose of 3 mg/kg for non‐small cell lung cancer, renal cell carcinoma, non‐Hodgkin lymphoma, head and neck cancer, and gastric cancer.^6^, ^7^, ^8^, ^9^, ^10^, ^11^, ^12^ In the United States, a fixed dose of 240 mg (calculated using 3 mg/kg for an average body weight of 77 kg) has been confirmed to be effective and safe in patients with malignant melanoma, non‐small cell lung cancer, and renal cell carcinoma and is being used in clinical practice.^1^ In Japan, a fixed dose (240 mg) of nivolumab for malignant pleural mesothelioma treatment was approved in August 2018. This approval was based on the results of a clinical study in patients with malignant pleural mesothelioma and a population pharmacokinetic analysis of the exposure dose, efficacy, and safety of nivolumab.^13^, ^14^

In a previous study, the weight of the target patients ranged from 34 to 180 kg, with 5% weighing less than 50 kg and 6% weighing more than 110 kg.^1^ For the general Japanese population, the average weight for males is 65.1 kg and the average weight for females is 52.1 kg for individuals aged 20 years and older, based on a 2015 report by the Japanese Statistics Bureau, Ministry of Internal Affairs and Communications. Patients with gastric cancer may lose weight due to symptoms, such as anorexia and nausea. Hence, the average weight of Japanese patients with gastric cancer is expected to be lower than that reported in previous studies, and it is not clear whether the drug can be administered safely as in previous studies. Nivolumab dose studies have only been conducted in patients with non‐small cell lung cancer, melanoma, and renal cell carcinoma, and there are no data for patients with gastric cancer. In addition, Japanese people tend to have a lower body size or weight than people from other nations. Differences in physique may result in differences in dosage and increased incidence and severity of side effects when drugs are administered at a fixed dose. There is also a possibility that side effects may differ depending on racial differences, increasing the urgency of evaluating the safety of different doses in Japanese patients. Therefore, we investigated the safety of nivolumab in Japanese patients with gastric cancer.

## 2 METHODS

This study was conducted in accordance with the tenets of the Declaration of Helsinki, and was reviewed and approved by the Clinical Research Ethics Review Committee of the Cancer Institute Hospital (approval No. 2019‐1110). As this was a retrospective study, informed consent was not sought from the patients. All patient data were anonymized and deidentified before analysis.

We reviewed the medical records of patients with gastric cancer for whom nivolumab treatment was initiated at our hospital between September 2017 and August 2019. Patients who had previously received immune checkpoint inhibitors and those who received both 3 mg/kg and fixed doses of nivolumab were excluded from the study.

### 2.1 Treatment and examination

Nivolumab was administered every 2 weeks until treatment discontinuation or the end of follow‐up, as previously described.^8^ All laboratory data, Eastern Cooperative Oncology Group performance status data, and medication history data were collected at our institution, and the follow‐up period ended on January 31, 2020.

Surviving patients were confirmed on the last follow‐up date. We defined immune‐related adverse events (irAEs) as events with an immunological rationale requiring frequent monitoring and possible intervention with immunosuppressive therapy. These included endocrine, hepatic, cutaneous, lymphatic, neurological, gastrointestinal, renal, pulmonary, pancreatic, and optic irAEs. We did not consider symptomatic factors with transient abnormal laboratory values, such as elevations in the levels of alanine aminotransferase, aspartate aminotransferase, and thyroid‐stimulating hormone, as irAEs. All adverse events were graded according to the Common Terminology Criteria for Adverse Events, version 4.0.

### 2.2 Frequency of irAE occurrence

The study endpoints were the incidence of Grade 2 or higher irAEs and the number of patients who discontinued nivolumab due to adverse events in the conventional‐ and fixed‐dose groups. The study endpoints were based on Grade 2 irAEs as these irAEs would result in nivolumab withdrawal.

### 2.3 Statistical analysis

The differences in patient background, irAEs grading (Grade 2 or higher), and frequency of irAEs between the groups were analyzed using Mann–Whitney *U* test and Fisher's exact test. Statistical significance was set at *p* < 0.05. Statistical analyses were performed using JMP version 13 software (SAS Institute Japan).

## 3 RESULTS

### 3.1 Patient characteristics

**Figure 1:**
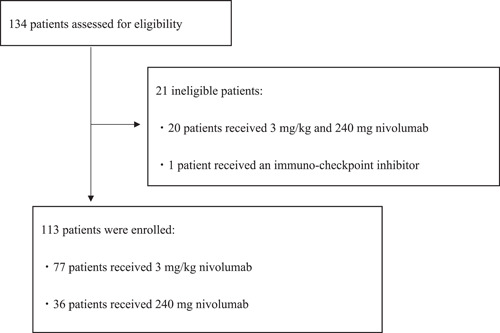
Patient selection process. Between September 2017 and August 2019, we screened 134 patients for eligibility; among them, 113 had received 3 mg/kg (*n* = 77) or 240 mg (*n* = 36) nivolumab. Twenty patients who had received both 3 mg/kg and fixed doses and one patient who had previously received immune checkpoint inhibitors were excluded.

Between September 2017 and August 2019, we screened 134 patients for eligibility; among them, 113 had received 3 mg/kg (*n* = 77) or 240 mg (*n* = 36) nivolumab, as shown in Figure 1. Twenty patients who had received both 3 mg/kg and fixed doses and one patient who had previously received immune checkpoint inhibitors were excluded. Table 1 shows patient demographics. No significant differences were observed between the groups in terms of sex, age, median weight, and history of autoimmune diseases. However, the number of patients who had received previous treatment regimens (*p* < 0.01) was significantly higher in the 3 mg/kg group than in the 240 mg group. The drugs used previously in the treatment of these patients included S‐1, cisplatin, irinotecan, trastuzumab, capecitabine, oxaliplatin, paclitaxel, ramucirumab, and docetaxel.

**Table 1:** Baseline characteristics of the patients

Characteristic	3 mg/kg (*n* = 77)	240 mg (*n* = 36)	*p*
Sex (male/female)	53/24	22/14	0.669*
Median age, years (range)	65 (33–84)	67 (37–84)	0.661**
ECOG‐PS (0/1/>2)	31/37/9	14/20/2	0.653*
Median body weight (kg) (range)	54.1 (35.7–80.0)	56.6 (37.0–84.7)	0.440**
History of autoimmune disease (# of patients)	2	0	1.000*
Previous treatment regimens (≥3/2)	33/44	5/31	<0.01*

Abbreviation: ECOG‐PS, Eastern Cooperative Oncology Group performance status scale.

* Fisher's exact test.

^**^ Mann–Whitney *U* test.

### 3.2 Frequency of irAE occurrence

The number of patients with irAEs of any grade was 23 (29.9%) and 7 (19.4%; *p* = 0.264) in the 3 mg/kg and 240 mg groups, respectively. Furthermore, the number of patients with Grade 2 or higher irAEs was 18 (23.3%) and 7 (19.4%) in the 3 mg/kg and 240 mg groups, respectively (*p* = 0.808; Table 2). These differences were not significant.

**Table 2:** Frequency of immune‐related adverse events (irAEs)

irAE grade	3 mg/kg *n* = 77	240 mg *n* = 36	*p*
All grades (%)	23 (29.9)	7 (19.4)	0.264*
Grade 2 or higher (%)	18 (23.3)	7 (19.4)	0.808*

* Fisher's exact test.

### 3.3 Observed irAEs according to category and grade

**Figure 2:**
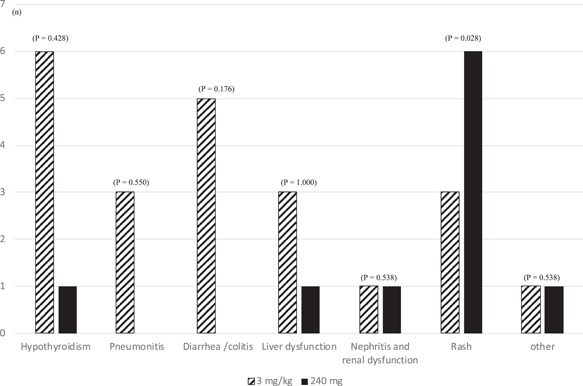
Observed immune‐related adverse events (irAEs) of Grade 2 or higher. Fisher's exact test was used for statistical analysis. The incidence of other irAEs of Grade 2 or higher was as follows: Six patients with hypothyroidism, three with pneumonitis, five with diarrhea/colitis, three with liver dysfunction, one with nephritis and renal dysfunction, three with rashes, and one with an adverse event designated as “other.” In the 240 mg group, the incidence of irAEs of Grade 2 or higher was as follows: One patient with hypothyroidism, one with liver dysfunction, one with nephritis and renal dysfunction, six with rashes, and one with an adverse event designated as “other.”

The incidence of other irAEs of Grade 2 or higher in the 3 mg/kg group was as follows: six patients with hypothyroidism, three with pneumonitis, five with diarrhea/colitis, three with liver dysfunction, one with nephritis and renal dysfunction, three with rashes, and one with an event designated as “other.” In the 240 mg group, the incidence of irAEs of Grade 2 or higher was as follows: One patient with hypothyroidism, one with liver dysfunction, one with nephritis and renal dysfunction, six with rashes, and one with an event designated as “other” (Figure 2). Hypothyroidism (*p* = 0.428), pneumonitis (*p* = 0.550), diarrhea/colitis (*p* = 0.176), liver dysfunction (*p* = 1.000), nephritis and renal dysfunction (*p* = 0.538), and other (*p* = 0.538) were not significantly related to the drug treatment groups. Furthermore, the frequency of rash (*p* = 0.028) was higher in the 240 mg group than in the 3 mg/kg group.

### 3.4 Reason for discontinuation

The reasons for treatment discontinuation in the 3 mg/kg group were as follows: pneumonia in two patients, skin disorder in one patient, liver disorder in two patients, diarrhea/colitis in one patient, and “other” in one patient. The reason for treatment discontinuation by one patient in the 240 mg group was idiopathic thrombocytopenic purpura (Table 3).

**Table 3:** Treatment discontinuation due to immune‐related adverse events

Reason	3 mg/kg *n* = 7	240 mg *n* = 1
Pneumonitis	2	0
Rash	1	0
Liver dysfunction	2	0
Diarrhea/colitis	1	0
Other	1	1

## 4 DISCUSSION

The study showed no significant difference in the cumulative occurrence of irAEs or the occurrence of grade 2 or higher irAEs between the 240 mg and 3 mg/kg treatment groups, suggesting that 240 mg of nivolumab is as safe as the 3 mg/kg dose. The dosages of several cytotoxic anticancer drugs are determined based on body surface area because of the proximity of effective and addiction ranges. Specifically, the dosage of molecularly targeted drugs is determined using body weight and body surface area, whereas drugs such as pertuzumab are administered at a constant dosage. For pertuzumab, the population pharmacokinetic model was constructed using data from Phase 1 of the TOC 2297g study and Phase 2 of the TOC 2689g and BO16934 studies. The model was then used to determine the fixed and weight‐equivalent dose of 840 mg as the initial dose and a fixed dose of 420 mg as the maintenance dose at 3‐week intervals. The steady‐state trough concentrations were simulated using either a fixed dose or a dose based on body surface area. Considering that 90% of patients achieved a target serum drug concentration of 20 μg/ml or higher, with either method of administration, the fixed dose was approved as the clinical dose.^15^, ^16^, ^17^, ^18^ The nivolumab fixed dose was approved after its safety was found to be comparable with that of weight‐based doses, which were initially approved based on population pharmacokinetics and dose/exposure‐response analyses in patients with malignant melanoma, non‐small cell lung cancer, and renal cancer.^1^ The median weight of patients with gastric cancer in this study was 54.1 and 56.6 kg in the 3 mg/kg and 240 mg groups, respectively (Table 1), which is approximately 20 kg lower than the weight for which the dose of nivolumab was initially calculated (77 kg). It is also significantly lower than the national average weight of Japanese men (65.1), indicating that the weight of patients with gastric cancer is lower than the national average weight.

In this study, 35% of the patients weighed less than 50 kg, compared with fewer than 5% of the patients who weighed less than 50 kg in a previous study that determined the fixed dose of 240 mg. However, no significant difference was observed in the occurrence of total or Grade 2 or higher irAEs (Table 2). In the global phase 3 study, the incidence of irAEs of all grades was 42.7%, compared with 29.9% and 19.4% in the 3 mg/kg and 240 mg groups, respectively.^8^ Specifically, the incidence of grade 2 or higher irAEs was 23.3% in the 3 mg/kg group and 19.4% in the 240 mg group, whereas the incidence of Grade 3–4 irAEs was 11.5% in the global phase 3 trial. However, as Grade 2 irAEs were included in this study, an accurate comparison with the findings of the global phase 3 trial cannot be made. Although this incidence differs from the findings of previous studies in patients with melanoma, non‐small lung cancer, and renal cancer, the dosage used in this study is nonetheless considered safe as the incidence of adverse events was lower than that in the global phase 3 trials and tended to be lower in the 240 mg group than in the 3 mg/kg group.^1^ Moreover, the MTD of nivolumab was 10 mg/kg in a previous study, with the maximum blood concentration achieved at 3 mg/kg, suggesting that fixed‐dose nivolumab could be safely administered to low‐weight patients with gastric cancer. Therefore, the 240 mg dose of nivolumab is considered as safe as the 3 mg/kg dose in these patients.

We could not compare the groups owing to the differences in patient characteristics. As shown in Table 3, although the results of this study differed in severity from those of the global phase 3 study, there was a similar trend of increased incidence of diarrhea, colitis, hypothyroidism, interstitial pneumonia, and hepatic dysfunction in the 3 mg/kg group.^8^ Specifically, in the 240 mg group, the incidence of skin symptoms was 16.7% for all grades. We also observed that the type of irAE differed with drug dosage; the 240 mg group presented with rashes more frequently than the 3 mg/kg group. The average weight of the group that developed rashes was 58.4 kg, and the average weight of the group that did not develop a rash was 54.4 kg. As the body weights were similar, it is possible, even if unlikely, that the difference in dosage affected the results, but the details of this difference are unknown.

There were some limitations to this study, including its retrospective design, small number of patients (in the 240 mg group), and short observation period (in the 240 mg group). The median duration of treatment was 42 days for the 3 mg/kg group and 56 days for the 240 mg group, and the median follow‐up intervals were 124 and 284 days, respectively. Six patients (16.7%) in the 240 mg group did not complete the treatment; however, as the incidence of irAEs was high in the early stages of the administration, and none of the patients developed irAEs after the end of treatment in the 3 mg/kg group, we believe that the effect on the results is small. Overall, considering that there are no previous studies on low‐weight patients with gastric cancer treated with a fixed dose of 240 mg, we believe that this safety assessment is significant. Correlation analysis between variables alone is not sufficient to draw conclusions from this study, and the results need to be confirmed in future studies involving more patients and more robust statistical analysis. In the next study, we plan to increase the number of patients and conduct multivariate analysis; therefore, we conducted this study in an exploratory manner to extract important factors for the subsequent study.

## 5 CONCLUSION

The administration of nivolumab at a fixed dose of 240 mg to patients with gastric cancer is as safe as the weight‐based 3 mg/kg dose, based on the findings from a small group of patients. To the best of our knowledge, this is the first report comparing the safety of nivolumab at 240 and 3 mg/kg in Japanese patients with gastric cancer, and we believe these findings will be useful for future studies.

## AUTHOR CONTRIBUTIONS

**Yusuke Iikura and Takeshi Aoyama**: Conceptualization. **Yusuke Iikura and Makoto Hiraide**: Formal analysis. **Takeru Wakatsuki, Izuma Nakayama, Mariko Ogura, Akira Ooki, Daisuke Takahari, Keisho Chin, Kensei Yamaguchi, and Toshihiro Hama**: Writing – review and editing. **Yusuke Iikura and Takeshi Aoyama**: Writing – original draft.

## CONFLICTS OF INTEREST

Kensei Yamaguchi has received speaking honoraria from Merck Serono, Takeda Pharmaceutical, Taiho Pharmaceutical, Chugai Pharmaceutical, Ono Pharmaceutical, Eli Lilly, Yakult, Sanofi, Bristol‐Myers Squibb, and Bayer, and research grants from Ono Pharmaceutical, MSD, Taiho Pharmaceutical, Sumitomo Dainippon Pharma, Eli Lilly, Daiichi Sankyo, Yakult, and Gilead Sciences. He also plays consulting and advisory roles for Bristol‐Myers Squibb. The other authors declare no conflict of interest.

## TRANSPARENCY STATEMENT

The corresponding author confirms that the manuscript is an honest, accurate, and transparent account of the study being reported, no important aspects of the study have been omitted, and any discrepancies from the study as planned have been explained.

## Data Availability

The data that support the findings of this study are available from the corresponding author upon reasonable request.
